# Bridging the Compatibility Gap in Revision Hip Arthroplasty with 14/16 Tapers: Long-Term Outcomes of the Bioball™ System

**DOI:** 10.3390/jcm15020771

**Published:** 2026-01-17

**Authors:** Marek Drobniewski, Bartosz Gonera, Łukasz Olewnik, Adam Borowski, Kacper Ruzik, George Triantafyllou, Andrzej Borowski

**Affiliations:** 1 Clinic of Orthopaedic and Paediatric Orthopaedics, Medical University of Lodz, 90-419 Lodz, Poland; marek.drobniewski@umed.lodz.pl (M.D.); andrzej.borowski@umed.lodz.pl (A.B.); 2 Department of Clinical Anatomy, Masovian Academy in Płock, 09-402 Płock, Poland; 3 Clinic of Orthopaedic, Medical University of Warsaw, 02-091 Warsaw, Poland; 4 Department of Anatomy, School of Medicine, National and Kapodistrian University of Athens, Goudi, 12462 Athens, Greece; georgerose406@gmail.com

**Keywords:** revision hip arthroplasty, Bioball System, femoral taper, offset restoration, stem retention

## Abstract

**Purpose:** Revision total hip arthroplasty (RTHA) in the presence of a well-fixed femoral stem is associated with increased risk, as stem removal often results in bone loss, prolonged operative time, and greater blood loss. This problem is particularly relevant for older implants with a 14/16 taper, which is incompatible with most modern femoral heads. The Bioball™ System, a modular head–neck adapter, allows for acetabular or head-only revision while preserving the femoral stem. This study aimed to evaluate long-term clinical and radiological outcomes of RTHA using the Bioball™ System in patients with 14/16 tapers. **Methods:** A total of 38 patients (23 women, 15 men; mean age 73.5 years) met the inclusion criteria. All procedures were carried out with a well-fixed femoral stem and a 14/16 taper. Revisions were limited to exchange of the acetabular component, liner, or both, avoiding stem removal. The primary indication was acetabular cup loosening (*n* = 29, 76.3%); liner-only exchange was performed in 9 patients (23.7%). Clinical outcomes were assessed using the modified Merle d’Aubigné and Postel (MAP) score, and radiological evaluation focused on fixation, migration, and loosening. Mean follow-up was 8.44 years. **Results:** Both the acetabular component and liner were replaced in 76.3% of patients, while 23.7% underwent liner and head exchange only. Longer adapter sizes were most frequently used, and a 7.5° offset adapter was applied in 57.9% of cases. The modified MAP score improved by a mean of 5.7 points (*p* < 0.05), and VAS pain scores decreased from 7.4 to 2.6 (*p* < 0.05). No radiological signs of loosening were observed at final follow-up. **Conclusions:** The Bioball™ System enables effective restoration of hip stability and offset without femoral stem removal, offering favorable long-term clinical and radiological outcomes in revisions involving older 14/16 tapers.

## 1. Introduction

The increasing demand for total hip arthroplasty (THA), especially among multimorbid elderly patients, presents a growing challenge for healthcare systems globally, with a significant risk of postoperative complications such as dislocation, infection, periprosthetic fractures, and bleeding [1]. Revision surgeries are frequently necessitated by factors like infection, aseptic loosening, mechanical failure, and suboptimal restoration of femoral offset, which can destabilize hip biomechanics, reducing abductor function and leading to functional impairments [2]. The revision procedure itself, especially when removing well-fixed femoral components, carries risks of substantial morbidity, including blood loss, infection, and prolonged operative time [3,4].

Over the past few decades, the design of femoral stem tapers in THA has undergone substantial modification. Earlier generations of implants commonly utilized a 14/16 mm taper geometry, providing a robust connection between the stem and the femoral head. However, starting in the 1990s, implant manufacturers progressively adopted smaller taper designs such as 12/14 mm, primarily to facilitate the use of smaller femoral heads and thereby increase the achievable range of motion and reduce the risk of impingement [5]. As described by Morlock et al. [6], this downsizing also reduced the contact area and bending stiffness of the taper junction, which later contributed to increased susceptibility to fretting and corrosion phenomena. Importantly, this design evolution led to a compatibility gap in revision procedures, since most modern femoral heads are now produced exclusively for 12/14 mm tapers. Consequently, revision cases involving older but well-fixed 14/16 mm femoral stems pose a technical limitation, as standard replacement heads cannot be directly mounted on the existing taper.

Although the taper size is commonly described by nominal dimensions such as 12/14 or 14/16, it should be emphasized that these designations do not imply full interchangeability. As demonstrated by Müller et al. [7], even tapers sharing the same nominal size may differ in angle, length, surface finish, and microgeometry, which can affect fixation mechanics and corrosion susceptibility.

Because of these compatibility challenges, revision surgeries on older implants require dedicated solutions that allow for head or liner exchange while retaining a stable femoral stem. One such option is the Bioball™ System (Merete Medical, Berlin, Germany), a modular head–neck adapter that provides a versatile and less invasive alternative in revision THA. It enables orthopedic surgeons to preserve a well-fixed femoral component while revising only the acetabular or head element, thus minimizing surgical trauma and operative time. This titanium adapter system, available in various lengths (−3 mm to +21 mm, S to 5XL) and compatible with multiple Morse tapers (e.g., 12/14, 14/16, and V40), provides options for adjusting femoral offset, leg length, and anteversion intraoperatively. Additionally, it supports modular heads in cobalt–chromium and ceramic materials in diameters from 28 mm to 58 mm, making it versatile across different patient anatomies and prosthetic requirements.

Research indicates that the Bioball™ System can effectively restore femoral offset and enhance joint stability without necessitating femoral stem revision, offering an attractive solution particularly in elderly or ASA class III and IV patients with significant comorbidities [1,3,8]. Notably, the Bioball™ System has demonstrated reliable long-term outcomes, especially for hip instability, through adaptations that protect the femoral neck–head junction and maintain functional biomechanics [1,8]. Although several studies describe the Bioball™ System as a modular adapter enabling offset restoration and dislocation management, no published work has evaluated long-term clinical and radiographic outcomes exclusively in revision THA (RTHA) patients retaining a well-fixed 14/16 taper stem. Existing studies combine various taper geometries, shorter follow-up durations, or cases where stems were revised. Consequently, evidence is lacking for a clinically important but narrow population where the Bioball™ System may prevent unnecessary stem removal.

The present study addresses this gap by reporting the longest available follow-up (mean 8.44 years) of RTHA procedures limited to acetabular component or liner exchange using the Bioball™ System in a strictly defined cohort with legacy 14/16 tapers. This design allows for an evaluation of whether the system provides durable function and stability in situations where alternatives are limited and conventional head exchange is not feasible.

## 2. Materials and Methods

**Figure 1:**
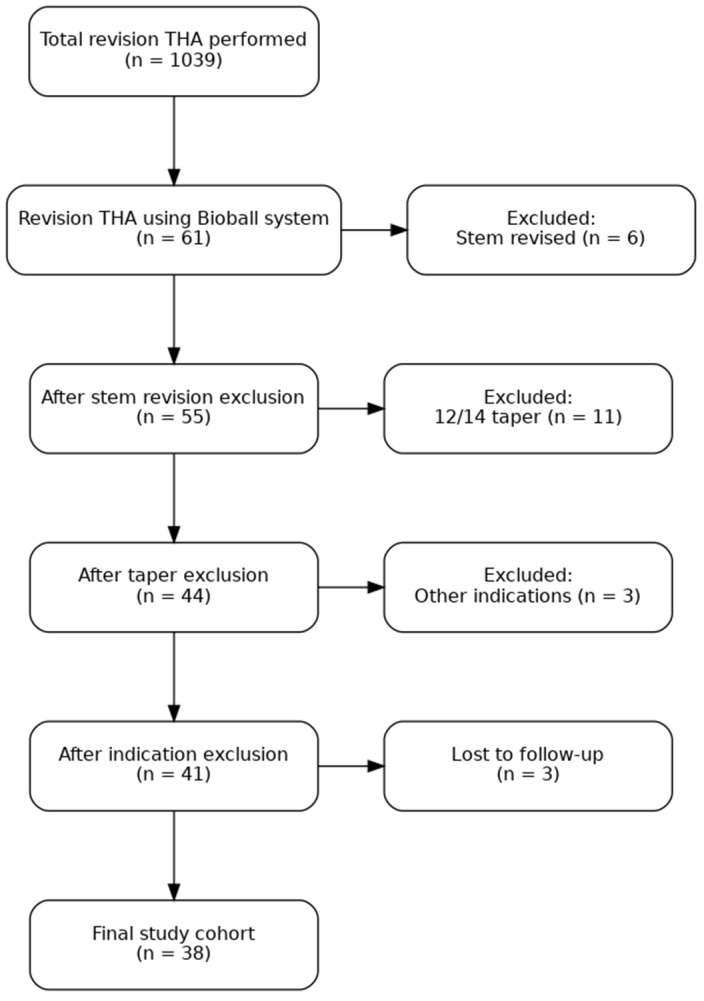
Flowchart of patient selection for the study. From 1039 RTHAs procedures performed, 61 involved the use of the Bioball System. Patients were excluded if the femoral stem was revised due to loosening (*n* = 6), if a 12/14 taper stem was used (*n* = 11), or if revision was performed for reasons other than aseptic loosening, including septic or post-traumatic causes (*n* = 3). Three patients were lost to follow-up, resulting in a final cohort of 38 patients.

Since 1974, our center has been performing THA. Currently, we conduct over 450 primary hip arthroplasties and approximately 90 revision procedures annually. This retrospective study analyzed patient data from 2008 to 2020, encompassing a total of 1039 revision THA procedures. Among these, 61 revisions involved the use of the Bioball™ system, limited to exchange of the acetabular component or liner, with a stable femoral stem featuring a 14/16 taper. We excluded 6 cases in which the femoral stem was also revised due to loosening, 11 cases involving stems with a 12/14 taper, and 3 cases in which revision was performed for reasons other than aseptic loosening (septic or post-traumatic). Three more patients did not return for subsequent follow-up. The final study cohort comprised 38 patients (Figure 1).

Among the patients treated, 23 were women (60.3%) and 15 were men (39.7%). The right hip was operated on in 16 cases (42.1%) and the left hip in 22 cases (57.9%). The average patient age at the time of surgery was 64.81 years, ranging from 24 to 86 years (SD: 12.21 years, Mdn: 66.5 years). The average follow-up period after revision surgery was 3082 days (8.44 years), SD: 1041.5 days, Mdn: 2872 days. All surgical procedures were performed by single senior orthopedic surgeon with more than 20 years of experience in arthroplasty.

In the study cohort, the Exception stem and L-Cup acetabular components (Biomet, Warsaw, Poland) were most commonly used; later, a press-fit Rimcup acetabular component (Biomet) was also utilized. Additional acetabular components included the Screwcup (BBraun/Aesculap). In 84% of cases, the implants were fixed to the bony bed using a cementless technique.

Radiological evaluation was integral to preoperative planning and follow-up assessments. For each case, anteroposterior and axial X-rays of the operated hip were obtained. Prosthesis positioning, acetabular and femoral component integration into bone tissue, and the presence and extent of heterotopic ossification were assessed. Migration of the acetabular component in the horizontal, vertical, and angular planes was assessed according to the DeLee and Charnley three-zone classification [9]. Routine imaging included a KingMark™ dual calibration ball system, serving as the reference for individual magnification factor determination. Digital templating was carried out in accordance with the description provided by Bono et al. [10,11], using TraumaCad™ software (Version: TraumaCad Neo, BrainLab, Chicago, IL, USA). Femoral offset was defined as the distance from the center of femoral head rotation to a line bisecting the femur’s long axis. Stem integration was analyzed using the Gruen and Moreland classifications [12,13], including evaluation of axial positioning within the femoral metaphysis, vertical migration, bone resorption and hypertrophy, bone density, and intraosseous and periosteal ossification identified across the seven defined zones. Cup inclination and cup anteversion were assessed according to established protocols. Radiographic evaluations were performed independently by two observers (A.B., Ł.O.) with more than 15 years of experience in orthopedic imaging diagnostics, both of whom are authors of numerous peer-reviewed publications in orthopedics and radiology; one is a senior orthopedic surgeon and the other an anatomist. Inter-observer agreement for radiographic parameters exceeded 0.90 (ICC), reflecting excellent reproducibility.

All procedures were performed under epidural anesthesia with an anterolateral approach, without greater trochanter osteotomy. In the cases where acetabulum was revised the acetabular component was placed within the Lewinnek safe zone [14], with typical acetabular anteversion not exceeding 15° and stem antetorsion between 5 and 10 most cases, the acetabular component was located in the anatomical true acetabular region. Polyethylene or ceramic acetabular inserts were used, with options including symmetrical (P-M) or asymmetrical 10° (Munich/Plasmacup) designs or fully ceramic (Mittelmeier) components. Additionally, the femoral stem was intraoperatively assessed in every case, with meticulous exclusion of any signs of loosening of damage.

Standard antibiotic and anticoagulant prophylaxis, in line with current epidemiological guidelines, was provided perioperatively. Rehabilitation commenced on the first postoperative day, and following removal of the Redon drain, patients were mobilized and trained to bear weight according to pain tolerance. Further rehabilitative exercises were introduced over the following days.

A control group as not included by design. The objective of this study was not to compare Bioball™-assisted revisions with conventional femoral stem revision procedures. These represent fundamentally different surgical interventions with distinct indications, levels of invasiveness, complication profiles, and expected recovery trajectories. The aim was to evaluate whether the Bioball™ System can serve as a clinically meaningful alternative in the narrow but important scenario where the femoral stem is well-fixed but incompatible with modern femoral heads due to a 14/16 taper. Thus, this cohort reflects a unique clinical subset in which unnecessary stem removal may be avoided.

After hospital discharge, follow-ups were scheduled at three, six, and twelve months, and annually thereafter. Clinical outcomes were assessed by the same surgeon at each follow-up, according to the Merle d’Aubigné and Postel classification, as modified by Charnley (Modified MAP) [15], to score pain, gait, and assessment of passive hip motion using a 1-to-6 scale per category (total possible score: 18). Pain was also measured using a ten-point Visual Analog Scale (VAS) [16], with “0” indicating no pain and “10” indicating the worst possible pain. Implant survival probability was planned to be analyzed using the Kaplan–Meier estimator [17], however we have experienced no components loosening after the revision surgery. Patients were followed from the date of revision until death or outcome, whichever occurred first. All individuals enrolled in the study provided informed consent.

The study was conducted in accordance with the Declaration of Helsinki and was approved by the Ethics Committee of the Medical University of Łódź (No. RNN/195/24/KE; approved on 10 September 2024).

### Statistical Analysis

Statistical analysis was performed with IBM Statistics for MacOS IBM SPSS Statistics for MacOS, Version 29 (IBM Corp., Armonk, NY, USA). Nominal data between unpaired observations were compared using the Chi-square test, while McNemar’s test was applied for paired observations. Normality was assessed with the Shapiro–Wilk test. A *p*-value < 0.05 was considered statistically significant. Continuous variables were analyzed according to data characteristics. Unpaired data were assessed using an independent *t*-test when normality assumptions were met; otherwise, the Mann–Whitney U test was applied. Paired data were analyzed with a paired *t*-test when normally distributed. Comparisons among more than two groups were performed using one-way ANOVA for normally distributed data or the Kruskal–Wallis test when normality was not satisfied.. The aim of the analysis was to investigate the association between patient gender and the extent of the procedure performed (i.e., liner-only replacement vs. replacement involving both the acetabular cup and liner). Additionally, it examined whether specific prosthesis head sizes and offset values were more frequently used for different procedure types.

## 3. Results

Patients had an average age of 64.81 years (SD: 12.21 years, Mdn: 66.5 years). The study group included 23 women and 15 men. The primary cause for the initial THA was secondary osteoarthritis due to idiopathic osteoarthritis in 21 patients (55.3%), developmental dysplasia of the hip in 13 patients (34.2%), and other causes in 4 patients (10.5%). The mean follow-up duration was 3082 days (Mdn: 2872 days, SD: 1041.5 days). Four patients passed away due to unrelated causes during the follow-up period, and their follow-up time was therefore excluded from the analysis. The mean Body Mass Index (BMI) of the patients was 27.85 (SD: 3.48, Mdn: 27.2). Among the study population, 6 out of the 38 patients had a history of primary prosthesis dislocation. All prior dislocation episodes, following closed reduction, did not require reoperation. The primary reason for revision surgery in the study group was acetabular cup loosening (*n* = 29, 76.31%), while only the liner was replaced in 9 patients (23.69%). No gender impact was observed (*p* = 0.310).

As regards the implants’ characteristics, the most used neck length was 2XL (+10.5 mm) (28.95% of cases (*n* = 11)), the least however was L (+3.5 mm) (2.63% of cases (*n* = 1)). The most frequently used head sizes was 32 mm (71.05% of patients (*n* = 27)), size 36 mm was used only in 2 cases (5.26% of cases). The size was not statistically significant between the procedure type (*p* = 0.060). The 7.5° offset adapter was used in 57.90% (*n* = 22) of patients. The offset values were not affected by the procedure type (*p* = 0.480). All details are summarized in Table 1.

As expected, preoperative clinical and radiological assessments were poor in all patients. At a mean follow-up of 8.44 years postoperatively, the final results, assessed using the modified Merle d’Aubigné and Postel (MAP) classification [15], were as follows: an excellent outcome was not observed in any case (0%), good outcome in 14 cases (36.84%), and fair outcome in 24 cases (63.16%). A poor outcome was not observed in any case (0%). The details mentioned above are summarized in Table 2. The modified MAP score demonstrated a mean increase of 5.7 points, reaching statistical significance (*p* < 0.05). The Modified MAP score was distributed the same between the different procedure types (*p* = 0.404).

Postoperative patient-reported outcomes were markedly superior to the objective results assessed using the modified MAP classification. The most pronounced improvements were observed in pain reduction and range of motion, leading to improved hip function and greater overall patient satisfaction. As expected, the most favorable outcomes were achieved in the group undergoing liner-only exchange. It should be noted that in the modified MAP classification, an “excellent outcome” indicates recovery comparable to that of an unaffected native hip. Crucially, no thigh pain, occasionally described after uncemented hip arthroplasty, was reported among the patients.

Pain intensity assessed using the Visual Analog Scale (VAS) [18], decreased from a preoperative mean of 7.4 points to 2.6 points postoperatively, representing a statistically significant reduction (*p* < 0.05).

During the entire follow-up period (mean 8.44 years), no postoperative complications were observed in the study cohort. Specifically, there were no cases of prosthetic dislocation, periprosthetic joint infection, aseptic loosening of any prosthetic component, persistent thigh pain, or need for re-revision surgery. Radiological follow-up revealed no signs of component migration, osteolysis, or mechanical failure. Furthermore, no clinical or radiographic findings suggestive of adverse reactions to metal debris (ARMD) were identified throughout the observation period.

A representative clinical case illustrating a typical indication for the use of the Bioball™ System is presented in Figure 1. The patient underwent revision total hip arthroplasty due to aseptic loosening of the acetabular component, while the femoral stem with a 14/16 taper remained well fixed. Preoperative radiographs demonstrate acetabular loosening with preserved femoral stem fixation, reflecting a common clinical scenario within the study cohort.

**Figure 2:**
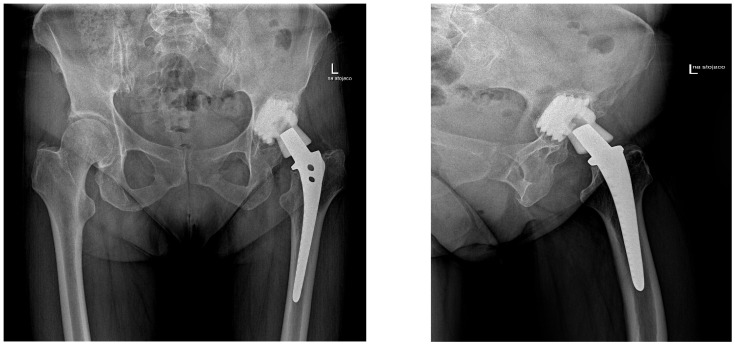
The figure presents a patient with aseptic loosening of the acetabular component, associated with extensive osteolysis and acetabular protrusion, while the femoral component (Mittelmeier stem) with a 14/16 taper remained well fixed. In addition, the patient presented with a 3 cm shortening of the left lower limb compared with the right side. L—Left.

**Figure 3:**
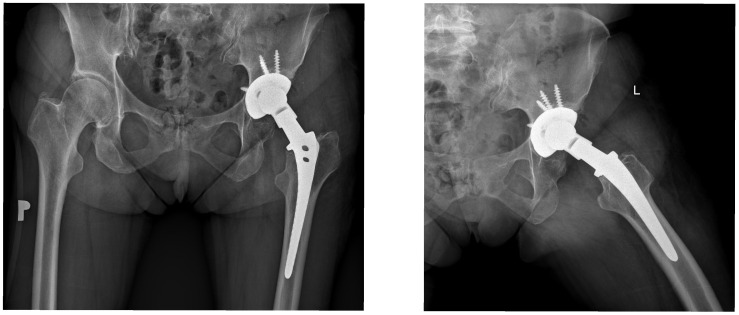
The figure presents the postoperative status of the same patient as in Figure 2. The acetabular component was revised using a 50 mm Bicontact Plasmacup MSC secured with four cancellous screws. The femoral stem–bone interface was inspected intraoperatively and the stem was found to be firmly fixed within the femoral canal. Due to a preoperative 3 cm shortening of the operated limb, a Merete BioBall™ 14/16 4XL head–neck adapter with a 7.5° offset and a 28 mm BioBall™ metal head were implanted. P—Right, L—Left.

Revision surgery was therefore limited to acetabular component exchange, and a Bioball™ head–neck adapter was used to restore femoral offset and limb length while avoiding femoral stem removal. Postoperative radiographs confirm correct positioning of the revised acetabular component and appropriate reconstruction of hip biomechanics (Figure 2 and Figure 3). At long-term follow-up, no radiological signs of loosening, migration, osteolysis, or other adverse findings were observed. This case exemplifies the typical surgical rationale, technical application, and durable outcome achieved with the Bioball™ System in patients with legacy 14/16 tapers.

All revised implants remained stable and functional until the final follow-up, corresponding to an implant survival rate of 100%. Although implant survival analysis using the Kaplan–Meier method was initially planned, it was not performed, as the absence of implant-related failure events would result in a flat survival curve without censoring, providing no additional informative value beyond the descriptive results presented.

## 4. Discussion

A unique and clinically significant aspect of our study is that all revision procedures were performed in patients with well-fixed femoral stems featuring a 14/16 taper. This taper geometry, once widely used in early generations of femoral stems, has been largely replaced by smaller 12/14 designs in modern arthroplasty systems. Consequently, surgeons are often confronted with a compatibility gap, as currently manufactured modular heads are no longer produced for 14/16 tapers. In such scenarios, a standard head exchange is technically impossible, often leading to unnecessary removal of a well-fixed femoral stem. The Bioball™ System provides an effective solution to this problem by offering an adapter-based interface that allows secure attachment of new modular heads to legacy 14/16 tapers. This approach enables true partial revision, limited to the acetabular component or head exchange, while preserving stable femoral fixation, thereby reducing operative time, intraoperative blood loss, and bone stock loss associated with stem removal. Furthermore, the adapter’s surface finish ensure mechanical stability while minimizing micromotion and corrosion risk at the taper junction.

While several studies have examined the Bioball™ System in mixed revision settings, no previous publication has reported long-term outcomes exclusively in patients retaining a well-fixed 14/16 taper stem. This distinction is critical: the incompatibility of modern heads with legacy tapers creates a unique therapeutic dilemma. If the femoral stem is well fixed but the taper geometry is obsolete, a surgeon may be forced to revise a stable stem purely due to lack of head compatibility. This represents preventable morbidity. By focusing solely on 14/16 tapers, our study directly addresses this underreported but increasingly relevant scenario as aging implants continue to reach revision age.

Another crucial aspect of achieving optimal function following THA is restoration of native femoral offset. While the importance of restoring femoral offset is well-established, the precise impact on functional outcomes remains a topic of ongoing debate. In recent years a lot has been debated about influence of femoral offset on strength, range of motion but also on pain sensation and quality of life [19,20,21,22]. Cassidy et al. [23] have shown that a significant decrease in femoral offset, particularly a reduction greater than 5 mm or 15%, can result in hip abductor weakness and reduced joint function. Similar findings have been reported by Bullen et al. [24], who found that a reduction greater than 20 mm in offset typically led to worse pain and motion scores. In our series, all patients were reoperated due to a cup loosening or liner wear which also decreased the femoral offset. After revision surgery with a head-neck adapter, all of the 38 examined patients experienced significant improvements in the Modified MAP scores [15] and we noted no dislocations of the endoprosthesis, demonstrating the potential benefit of restoring femoral offset to improve functional outcomes and address abductor weakness.

Stability in revision THA depends heavily on femoral offset, abductor tension, and jump distance. In our series, offset restoration using the Bioball™ System likely contributed to the absence of dislocations. Increasing offset enhances abductor muscle function, improves the joint reaction force vector, and increases the energy barrier for dislocation. Even in patients with prior dislocation episodes or dysplastic anatomy, no postoperative instability was noted, underscoring the biomechanical advantage of controlled offset restoration

The head-neck adapter has become an essential tool in revision surgeries, especially in cases where only partial hip revision is required. By retaining the original femoral stem and replacing the taper surface on the damaged cone, the adapter minimizes the need for more extensive revision procedures. Moreover, the addition of a 7.5° offset via the head-neck adapter can significantly improve hip stability, particularly in cases of recurrent dislocation, as highlighted by Kock et al. [1]. The utility of the adapter in primary THA, particularly in patients with a high risk of dislocation, has also been reported by Kock et al. [1]. In our series, we utilized the 7 different head-neck adapters in (from M to 5XL) to increase femoral neck length and also in 57.90% of cases special taper was used to adjust the center of rotation with a 7.5° offset. These applications demonstrate the versatility of the Bioball™ System in both complicated revision procedures.

Hoberg et al. [25]. examined retrospectively 95 consecutive patients revised with the Bioball adaptor system. 13 patients were lost to follow-up and a total of 82 patients were reviewed. Recurrent dislocations, acetabular component loosening and wear of the acetabular liner were indicated to be the most common indications for revision in the examined group. In their study satisfaction was assessed in a ternary fashion (i.e., whether satisfied, partly satisfied or not satisfied). They have noted mostly excellent results—approximately 90% of patients reported satisfaction after the revision surgery, whereas only 1% of patients in their cohort reported no satisfaction. Moreover the survival rate of the Bioball™ System accounted for 92.8% after 8.17 years. Interestingly they have reported 100% of survival rate at 2-years follow up. Similar results were obtained by Dabis et al. [26], who have reviewed the outcomes of 32 hip revision arthroplasty procedures using the Bioball™ System. During a minimum follow-up period of two years, two cases of recurrent dislocations of the artificial joint were reported following revision surgery, as well as one case of instability without dislocation of the prosthesis. In our study we have noted 100% survival rate after 8.44 years of follow-up and only good (36.84%) and fair (63.16%) results at the latest appointment.

Similar results to ours were obtained by Jack et al. [27], who studied the outcomes of ceramic-on-ceramic articulation in isolated acetabular component revision procedures of THA. In their study group, a total of 165 revision procedures were performed, with the Bioball™ System used in 75 cases. During a mean follow-up period of 8.3 years, they achieved a biofunctional rate of the acetabular component at 96.6%. An important challenge in revision hip arthroplasty is joint instability, which can result in recurrent dislocations. Woelfle et al. [28] have analyzed the outcomes of 44 hip revision surgeries using the Bioball™ System. Over a four-year follow-up period, only three cases of recurrent dislocation of the artificial joint were reported. However, due to the advanced age of the patients and the coexistence of other chronic medical conditions, the average clinical outcome (HHS = 54) was deemed relatively poor. In their conclusions, the authors emphasized the positive role of the Bioball™ System in reducing postoperative leg length discrepancies and highlighted its beneficial impact on restoring proper biomechanical conditions of the operated hip joint (offset). In our study, despite a high rate of dislocations of the primary prosthesis (15.79%), no cases of prosthetic dislocation were observed following revision surgery throughout the entire follow-up period. Interestingly, the MAP score in patients with episodes of primary prosthesis dislocation, after revision surgery was nearly identical to that of patients with no episodes of prosthesis dislocation (14.19 vs. 14.39, respectively). The comparison yielded a *p*-value of 0.574, indicating no statistically significant differences between these groups.

Pautasso et al. [29] have performed comprehensive narrative review analyzing the outcomes of hip revision arthroplasty using the BioBall^®^ adapter in various clinical scenarios. It included data from multiple studies and the timeframe was limited between 1 January 2000 and 1 December 2022, with groups ranging from 32 to 194 patients. Follow-up periods varied, and dislocation rates ranged from 5.2% to 15%. Patient satisfaction was mostly high, with studies reporting Harris Hip Scores averaging 80.9 and satisfaction rates of up to 89%. Complications were rare, and the ones reported were fatigue fractures of the neck adapter, third body wear, stem neck fracture, ceramic head fracture and disassembly of the system components. None of the described complications have occurred in our study.

Patients with hip dysplasia, characterized by reduced femoral offset, present a unique challenge during THA [30]. If not carefully managed, correction of femoral offset in these patients can lead to an excessively increased offset. Cassidy et al. [23] reported that 22.6% of patients with a decreased offset preoperatively still had residual offset reduction postoperatively despite the use of extended offset stems. In our study, thirteen patients with congenital hip abnormalities presented with a diminished offset were included in the study. Despite anatomical abnormalities and difficulties the revision surgeries were successfully addressed with the Bioball™ System, reinforcing the adapter’s role in precise offset restoration, particularly in complex cases.

Corrosion, fretting, taper wear, and modular neck failures have been documented in the literature, particularly in systems with smaller cross-sections or mismatched taper geometries. Importantly, no such complications were observed in our cohort. All components remained radiographically stable with no measurable migration, osteolysis, or adverse mechanical changes. These findings suggest that, when used appropriately and in a well-fixed stem, the Bioball™ System does not inherently increase mechanical risk.

Our study, based on a retrospective analysis of 38 adult patients with a mean observation time of 3082 days, provides valuable insight into the effectiveness of the Bioball™ System in revision THA. Notably, we observed no dislocations or loosening of prosthetic components after the revision procedures, which further supports the reliability and stability of the Bioball™ System. However, the study is limited by its retrospective design and relatively small sample size; however, both constraints are inherent to the rare and highly specific patient subgroup examined. Another limitation is the lack of broader patient-reported outcome measures such as the Harris Hip Score or WOMAC, which were not routinely collected during the study period. Additionally, the management of periarticular soft tissues, such as gluteus medius tears, could have contributed to the observed improvements in functional outcomes. Future prospective studies incorporating detailed functional assessments and larger cohorts will help refine indications for use of the Bioball™ System.

## 5. Conclusions

In conclusion, the use of the Bioball™ System allows for achieving hip joint stability without the need to remove a stable femoral component. This substantially reduces the extent of the procedure, blood loss, and complications that may arise during stem removal. Importantly, it enables limitation of surgical invasiveness in cases where the femoral stem remains stable but the acetabular component or insert is loosened, provided that a 14/16 taper is present—a scenario that would not be feasible with the currently prevailing 12/14 head standard. Moreover, the Bioball™ System is a promising solution for addressing femoral offset restoration in both revision and primary THA. Our study demonstrates its effectiveness in improving clinical outcomes, particularly in patients with decreased femoral offset or those at risk of recurrent dislocation. Further research is required to validate these findings and refine the criteria for its use in diverse clinical scenarios.

## Figures and Tables

**Table 1:** Patient demographics and baseline characteristics.

Variable	Value
Age at revision surgery (years)	64.8 ± 12.2 (range 24–86)
Sex	23 female (60.3%), 15 male (39.7%)
BMI (kg/m^2^)	27.9 ± 3.5
Operated side	Right: 16 (42.1%), Left: 22 (57.9%)
Follow-up	3082 ± 1041 days (mean 8.44 years)
Primary indications to THA	Idiopathic osteoarthritis: 21 (55.3%) Developmental dysplasia: 13 (34.2%) Other causes: 4 (10.5%)
History of primary prosthesis dislocation	6 patients (15.8%)
Indication for revision surgery	Acetabular cup loosening: 29 (76.3%) Liner wear only: 9 (23.7%)
Extent of revision procedure	Cup + liner exchange: 29 (76.3%) Liner + head exchange only: 9 (23.7%)
Femoral head size	28 mm: 9 (23.7%) 32 mm: 27 (71.1%) 36 mm: 2 (5.3%)
Bioball™ neck length	M–5XL (most frequent: 2XL + 10.5 mm, *n* = 11, 28.9%)
Offset adapter (7.5°)	Used in 22 patients (57.9%)

**Table 2:** Final outcomes according to the Merle d’Aubigné and Postel classification, as modified by Charnley, were compared between the preoperative and postoperative groups using a paired *t*-test. A *p*-value < 0.05 was considered statistically significant.

Outcome Measure	Preoperative	Postoperative	Statistical Analysis (Paired *t*-Test)
Modified MAP score (points), mean ± SD	8.62 ± 1.10	14.29 ± 0.97	***p*** < 0.001; 95% CI: 5.12–6.24

## Data Availability

The raw data supporting the conclusions of this article will be made available by the authors on request.

## Abbreviations

RTHA—revision total hip arthroplasty; THA—total hip arthroplasty; VAS—Visual Analog Scale, BMI—The Body Mass Index, MAP—Merle d’Aubigné and Postel, modified by Charnley.
